# Integrated Ovarian circRNA, miRNA and mRNA Profiling Identifies Candidate ceRNA Relationships Associated with Late-Laying and Nesting Stages in Wanxi White Geese

**DOI:** 10.3390/genes17101227

**Published:** 2026-10-02

**Authors:** Kefeng Yang, Tongsheng Yang, Dexing Cui, Tao Jin, Man Ren, Qianqian Hu, Changsheng Jiang, Chunfang Zhao, Shenghe Li

**Affiliations:** 1 College of Animal Science, Anhui Science and Technology University, Chuzhou 233100, China; 15505672192@163.com (K.Y.); 19855656167@163.com (T.Y.); 17860233486@163.com (D.C.); yjs2024387@ahstu.edu.cn (T.J.); renman@ahstu.edu.cn (M.R.); huqq@ahstu.edu.cn (Q.H.); jiangcs@ahstu.edu.cn (C.J.); 2 Anhui Province Key Laboratory of Animal Nutritional Regulation and Health, Chuzhou 233100, China; 3 Anhui Engineering Technology Research Center of Pork Quality Control and Enhance, Chuzhou 233100, China; 4 Lu’an Academy of Agricultural Sciences, Lu’an 237008, China

**Keywords:** Wanxi white goose, late-laying, nesting, ovarian regression, circRNA, miRNA, ceRNA network

## Abstract

**Background**: The pronounced broodiness of Wanxi white geese is associated with rapid ovarian regression and reduced egg production and reproductive efficiency. However, the extent to which circRNAs influence this physiological transition by coordinating ceRNA interactions is not yet well understood. **Methods**: Here, we generated ovarian microRNA (miRNA) and circRNA expression profiles by RNA sequencing in late-laying (LLG) and nesting (NG) Wanxi white geese and measured serum reproductive hormones. **Results**: Compared with the late-laying stage, the nesting stage was characterised by pronounced ovarian atrophy. Serum concentrations of oestradiol (E2), progesterone (P4) and luteinising hormone (LH) were lower, whereas the prolactin (PRL) concentration was higher. Transcriptome analysis identified 156 circRNAs and 64 miRNAs that met the nominal differential expressed (DE) criteria. Enrichment analysis associated genes giving rise to DE circRNAs with membrane and junctional structures, cell adhesion, small GTPase regulation, ECM–receptor interaction, adherens junction, focal adhesion and metabolic processes. Conversely, putative targets of DE miRNAs were linked to AMPK, mTOR, TGF-β and Hippo pathways, together with oocyte meiosis. The integration of the RNA datasets and Pearson correlation analysis highlighted several putative ceRNA relationships associated with ovarian functional transition. During the late-laying period, bioinformatic predictions and expression correlations supported the predicted novel_circ_001280-miR-1-y-CPEB1 and novel_circ_005385-miR-187-x-BMP6 relationships involving genes associated with oocyte development and ovarian endocrine function. By contrast, during the nesting period, bioinformatic analyses supported a putative novel_circ_002435/novel_circ_007511-miR-135-x/z-FOXO3 relationship that may be associated with cellular quiescence, stress responses and follicular atresia. The expression profiles of these network components showed stage-associated correlations with circulating reproductive hormone concentrations. **Conclusions**: Together, these findings suggest an exploratory ceRNA landscape associated with ovarian functional remodelling in Wanxi white geese and identify RNA relationships that warrant further functional validation.

## 1. Introduction

The morphology and physiological activity of the avian ovary undergo marked, stage-dependent adaptations throughout the reproductive cycle [1]. Native to China, the Wanxi white goose is a commercially valuable breed, distinguished by premium down and desirable meat-production characteristics. However, its pronounced maternal incubation behaviour referred as the nesting stage is accompanied by a substantial functional transition of the ovary from the late-laying stage to the nesting stage. The onset of broodiness alters the hypothalamic–pituitary–ovarian (HPO) axis, leading to extensive follicular atresia, cessation of ovulation and a marked decline in egg production [2]. It is a coordinated behavioural, endocrine and reproductive organ state accompanied by persistent nest attendance and reduced feeding and activity, which collectively contribute to the interruption of egg laying and delay the recovery of reproductive activity [3]. These changes reduce reproductive efficiency and erode the profitability of poultry production. It therefore remains an important but challenging objective to elucidate the biological basis of broodiness in poultry reproductive biology and breeding. Current evidence suggests that fluctuations in reproductive hormones, including prolactin, oestradiol and progesterone, act together with complex post-transcriptional regulatory networks to coordinate ovarian remodelling during this reproductive transition [4,5]. RNA molecules without protein-coding capacity regulate reproduction in both normal and pathological states. Among them, miRNAs [6] and lncRNAs [7] modulate follicle development, apoptosis and proliferation in granulosa cells, and steroidogenesis. Although the roles of miRNAs and lncRNAs in follicular development and reproductive endocrine regulation have been extensively studied, other classes, particularly circRNAs, remain comparatively understudied. These molecules form when back-splicing covalently joins the ends of precursor transcripts, creating closed RNA circles without protein-coding capacity. Because their closed structure contains neither a 5’ cap nor a 3’ poly(A) tail, circRNAs resist exonuclease-mediated degradation, remain more stable than linear transcripts and often exhibit tissue-specific expression. Some evidence indicates that circRNAs can act as miRNA sponges within ceRNA networks [8,9,10], thereby influencing apoptosis, reproductive development and granulosa-cell differentiation [11]. By binding specific miRNAs, circRNAs may relieve miRNA-mediated repression of downstream mRNAs, with potential consequences for ovarian endocrine function and follicular atresia [12,13].

However, research on non-coding RNAs in poultry has focused mainly on muscle growth and fat deposition. The circRNAs involved in reproductive transitions in waterfowl, particularly during the nesting stage, remain poorly characterised, and their regulatory roles in ovarian regression and remodelling are not well understood. Recent studies have begun to examine ovarian circRNA expression in geese. Stage-dependent circRNA profiles and predicted circRNA-miRNA regulatory relationships have been reported in Yili goose ovaries across the pre-laying, laying and ceased-laying periods [14]. We previously described the histological features and the mRNA transcriptome of the Wanxi white goose ovary at these two stages [7]; that work, however, did not address non-coding RNA regulation, and the circRNA and miRNA layers of this transition therefore remain unexamined. We applied high-throughput transcriptome sequencing (RNA-seq) to comprehensively profile ovarian RNA in Wanxi white geese sampled during the late-laying and nesting stages. Accordingly, we compared circRNA and miRNA profiles across the two reproductive stages to identify stage-dependent expression changes and characterise their dynamics during ovarian functional remodelling. We then integrated these datasets with the previously published mRNA profiles to infer a putative circRNA–miRNA–mRNA ceRNA network linked to the ovarian functional transition. To assess their functional relevance, predicted targets were tested for enrichment across Gene Ontology (GO) categories and Kyoto Encyclopedia of Genes and Genomes (KEGG) pathways. Together, these approaches enabled the prioritisation of RNA candidates and biological pathways potentially involved in the shift from late-laying to nesting. This framework enables systematic investigation of post-transcriptional regulation during the nesting stage and may guide future molecular breeding strategies to reduce broodiness and improve laying performance in waterfowl.

## 2. Materials and Methods

### 2.1. Experimental Animals and Sample Collection

The experimental cohort comprised 120 healthy 365-day-old female Wanxi white geese sourced from Anhui Science and Technology University’s waterfowl breeding flock in Dingyuan County, China. The 120 geese constituted the source population for sampling and the remaining 100 birds were not used for other sampling, experiments or measurements in this study. All birds were housed under uniform husbandry conditions. All birds were housed in the same building under identical management, received the same diet and had ad libitum access to water. The Anhui Provincial Laboratory Animal Ethics Committee reviewed and approved all animal procedures and protocols (approval no. AHSTULL2022016). Animal care and experimental use followed the relevant national standards governing laboratory-animal welfare. The late-laying group comprised females in the post-peak portion of the laying cycle, when egg production had begun to decline but had not ceased [15]. Females were assigned to the nesting group when broody behaviour persisted for at least four successive days and was accompanied by defensive behaviour around the nest and observable reductions in feeding and general activity [16]. At each reproductive stage, late laying or nesting, ten females were selected at random. Before sampling, food was withheld for 12 h, while drinking water remained freely available. Wing-vein blood was transferred to tubes designed for serum preparation. Centrifugation at 4 °C and 3000× *g* for 20 min yielded serum, which was promptly frozen at −80 °C pending hormone measurement. Of the ten females sampled for blood collection at each stage, three were randomly selected and euthanised by exsanguination. Ovarian tissues containing follicles at different developmental stages were collected for total RNA extraction.

### 2.2. Serum Hormone Measurements

Serum concentrations of PRL, E2, P4, AMH, LH and FSH were measured using commercial enzyme-linked immunosorbent assay (ELISA) kits (Nanjing Jiancheng Bioengineering Institute, Nanjing, China) according to the manufacturer’s instructions. All samples were assayed in triple on a single plate per hormone.

### 2.3. RNA Extraction, Library Preparation and Sequencing

Immediately upon collection, ovarian samples were immersed in liquid nitrogen for rapid cryopreservation and then maintained at −80 °C until processing. Total RNA was isolated with TRIzol (Invitrogen; Carlsbad, CA, USA). Concentration and purity were determined separately, integrity was checked, and preparations passing quality control proceeded to library construction. For mRNA profiling and circRNA detection, rRNA was removed before the residual RNA was fragmented. Random hexamers primed synthesis of the initial cDNA strand, while dUTP was incorporated during generation of the complementary strand. The cDNA products then underwent purification, end repair, 3’ adenylation and sequencing-adapter attachment. Following UNG treatment, fragment-size selection and PCR enrichment, the strand-specific libraries were processed with an Illumina HiSeq™ 4000 system (Illumina, Inc., San Diego, CA, USA). Small-RNA libraries were prepared from 18–30-nt RNA fragments recovered by polyacrylamide gel electrophoresis (PAGE). Adapters were attached sequentially to the 3’ and 5’ termini, and the ligated RNA was reverse-transcribed before PCR amplification. Products measuring approximately 140–160 bp were recovered by PAGE and resuspended in EB buffer. An Agilent 2100 Bioanalyzer (Agilent Technologies, Palo Alto, CA, USA)was used for quality assessment, whereas library concentrations were determined with an ABI StepOnePlus Real-Time PCR System (Applied Biosystems, Foster City, CA, USA). The small-RNA libraries were subsequently processed on the Illumina HiSeq X Ten platform (Illumina, Inc., San Diego, CA, USA).

### 2.4. Quality Filtering of Sequencing Reads and Reference-Genome Mapping

The raw sequencing datasets can be accessed through the Sequence Read Archive (SRA), a repository maintained by the National Center for Biotechnology Information (NCBI) [7]. Associated run accessions range from SRR30688733 to SRR30688738. Separate records for the circRNA and miRNA datasets span SRR40170502 to SRR40170513. The raw reads from the rRNA-depleted transcriptome libraries used for circRNA identification were filtered with fastp (v0.18.0). Reads were excluded when adapters were present, ambiguous bases exceeded 10%, or bases with Q scores ≤ 20 accounted for more than 50% of the sequence. Reads made entirely of adenine residues were removed during the same filtering step. Mapping to an rRNA reference was performed with Bowtie2 (v2.2.8), and all rRNA-associated reads were excluded. The retained paired-end reads were mapped with HISAT2 (v2.1.0) to the goose genome AnsCyg_PRJNA183603_v1.0 (Anser cygnoides; GCF_000971095.1). The --rna-strandness RF option was specified, while every other setting retained its default value. Mapped reads supported mRNA analysis, whereas unmapped reads were carried forward for circRNA identification. For every sample, the Q30 proportion, GC content, and mapping rate were recorded.

Initial processing of small-RNA reads removed low-quality sequences and those containing ambiguous nucleotides, missing 3’ adapters, or consisting solely of adapter sequence. Sequences containing poly(A) and those shorter than 18 nt were also excluded. The cleaned tags were compared with GenBank release 209.0 and Rfam release 11.0 to detect non-miRNA contaminants. Tags annotated as rRNA, scRNA, snoRNA, snRNA or tRNA were eliminated before genome mapping. Bowtie (v1.1.2) was then applied to map the retained tags against the Anser cygnoides reference assembly. Sequences matching miRBase entries were classified as previously known miRNAs. Potentially novel miRNAs were inferred from their genomic coordinates and characteristic hairpin structures. Downstream expression analysis used a unified set containing both known and putative novel miRNAs.

### 2.5. Identification and Annotation of circRNAs

Reads remaining unaligned after HISAT2 mapping against the reference genome were retained for circRNA discovery. For each retained read, 20-nt segments from the 5’ and 3’ termini served as anchors and were aligned separately with Bowtie2 in single-end mode. Anchor alignments from all samples were pooled and analysed using find_circ (v1) to identify reads spanning potential back-splice junctions. Candidate circRNAs were retained when they met all of the following criteria: a single unambiguous breakpoint; an anchor overlap of no more than 2 bp; no more than two mismatched bases; more than two unique supporting reads; a difference of more than 35 between the best and second-best alignment scores for at least one anchor; unique read support exceeding half the total number of samples, defined as n_uniq > int(n_samples/2); and a predicted circRNA length of less than 100 kb. Their host genes and genomic categories were annotated according to their genomic locations. The identified circRNAs were compared with circBase, and unmatched circRNAs were classified as novel. Because circBase contains no entries for Anser cygnoides, essentially all circRNAs reported here carry the “novel_circ” designation; this nomenclature reflects the absence of a goose circRNA reference rather than demonstrated experimental novelty. Back-splice junctions were identified computationally and were subsequently amplified with divergent primers during RT-qPCR validation.

### 2.6. Differential Expression and Functional Enrichment Analyses of circRNAs and miRNAs

RPM (reads per million mapped reads) served as the normalised abundance measure for circRNAs, whereas TPM (transcripts per million) was used for miRNAs. StringTie version 1.3.4 estimated mRNA expression in FPKM units (fragments per kilobase of transcript per million mapped reads). These normalised measurements supported expression summaries and visualisation, while raw count matrices were retained for differential-expression testing. Raw circRNA and miRNA counts were analysed with edgeR to identify DE transcripts. For the miRNA analysis, dispersion was estimated from three biological replicates per group. DE mRNAs were detected with DESeq2 under a negative-binomial model. For all three RNA classes, DE status required |log_2_FC| ≥ 1 and *p* < 0.05. Functional enrichment was assessed for the host genes associated with DE circRNAs, predicted targets of DE miRNAs, and DE mRNAs. GO and KEGG analyses were performed with clusterProfiler and KOBAS. Statistical significance for enriched terms and pathways was defined at *p* < 0.05.

### 2.7. Integrated Analysis of circRNAs, miRNAs and mRNAs and Construction of the ceRNA Network

Potential miRNA-Binding sites in circRNAs and mRNAs were independently predicted using miRanda (v3.3a) and TargetScan (v7.0) applied to custom goose sequence inputs, and only interactions predicted by both programs were retained. An exploratory ceRNA network was constructed from RNAs passing the nominal DE screen. miRNA-circRNA and miRNA–mRNA pairs with Spearman’s ρ < −0.7 and circRNA–mRNA pairs with Pearson’s *r*  > 0.9 were retained. Each circRNA–mRNA pair was required to share at least one predicted miRNA, with enrichment assessed using a cumulative hypergeometric test (*p* < 0.05). Candidate triplets were further prioritised when circRNA and miRNA expression changed in opposite directions, miRNA and mRNA expression changed oppositely, and circRNA and mRNA expression changed consistently between the LLG and NG stages. Pearson correlations based on normalised expression values were used as an additional consistency check. Target predictions, expression patterns, correlations and functional annotations were integrated to identify candidate ceRNA axes, which were visualised using Cytoscape (v3.5.0).

### 2.8. Validation by Quantitative Reverse Transcription PCR

Primer sets for mRNAs, circRNAs and miRNAs were generated with Primer Premier 5.0, circPrimer 2.0 and miRNA Design (v1.01), respectively. Supplementary Table S1 provides their complete sequences. Five DE mRNAs, five DE circRNAs and six DE miRNAs were selected for validation. Divergent primer pairs spanning the relevant back-splice junctions were used to amplify circRNAs. The RevertAid First Strand cDNA Synthesis Kit (Vazyme Biotech, Nanjing, China) was used to prepare cDNA templates for mRNA and circRNA assays. miRNA cDNA was generated with specific stem-loop primers and Vazyme’s miRNA 1st Strand cDNA Synthesis Kit (Nanjing, China). RT-qPCR reactions were run on an ABI 7500 Real-Time PCR System (Applied Biosystems, Foster City, CA, USA). The reaction mixture for mRNAs and circRNAs contained Power SYBR Green PCR Master Mix, whereas miRNA assays used miRNA Universal SYBR qPCR Master Mix from Vazyme. Three independent biological replicates from the same geese used for RNA sequencing were included per group, and each was tested in three technical reactions. β-actin was used to normalise mRNA and circRNA data, while miRNA measurements were normalised against U6. Relative abundance was derived using the 2^−ΔΔCt^ method.

### 2.9. Statistical Analysis

Unless otherwise stated, quantitative data are presented as the mean ± standard deviation (s.d.). Statistical analyses were performed using IBM SPSS Statistics v27.0. Differences between the LLG and NG groups were assessed using two-tailed independent-samples *t*-tests. For RT-qPCR data, triplicate technical Ct values were averaged for each biological replicate, and biological replicates were treated as independent experimental units. Pearson’s correlation coefficients were calculated to assess associations between RNA expression levels and serum reproductive hormone concentrations in the three birds per stage for which both datasets were available. All statistical tests were two-sided, and *p* < 0.05 was considered statistically significant.

## 3. Results

### 3.1. Ovarian Morphology and Serum Hormone Concentrations Across the Transition from Late-Laying to Nesting

**Figure 1:**
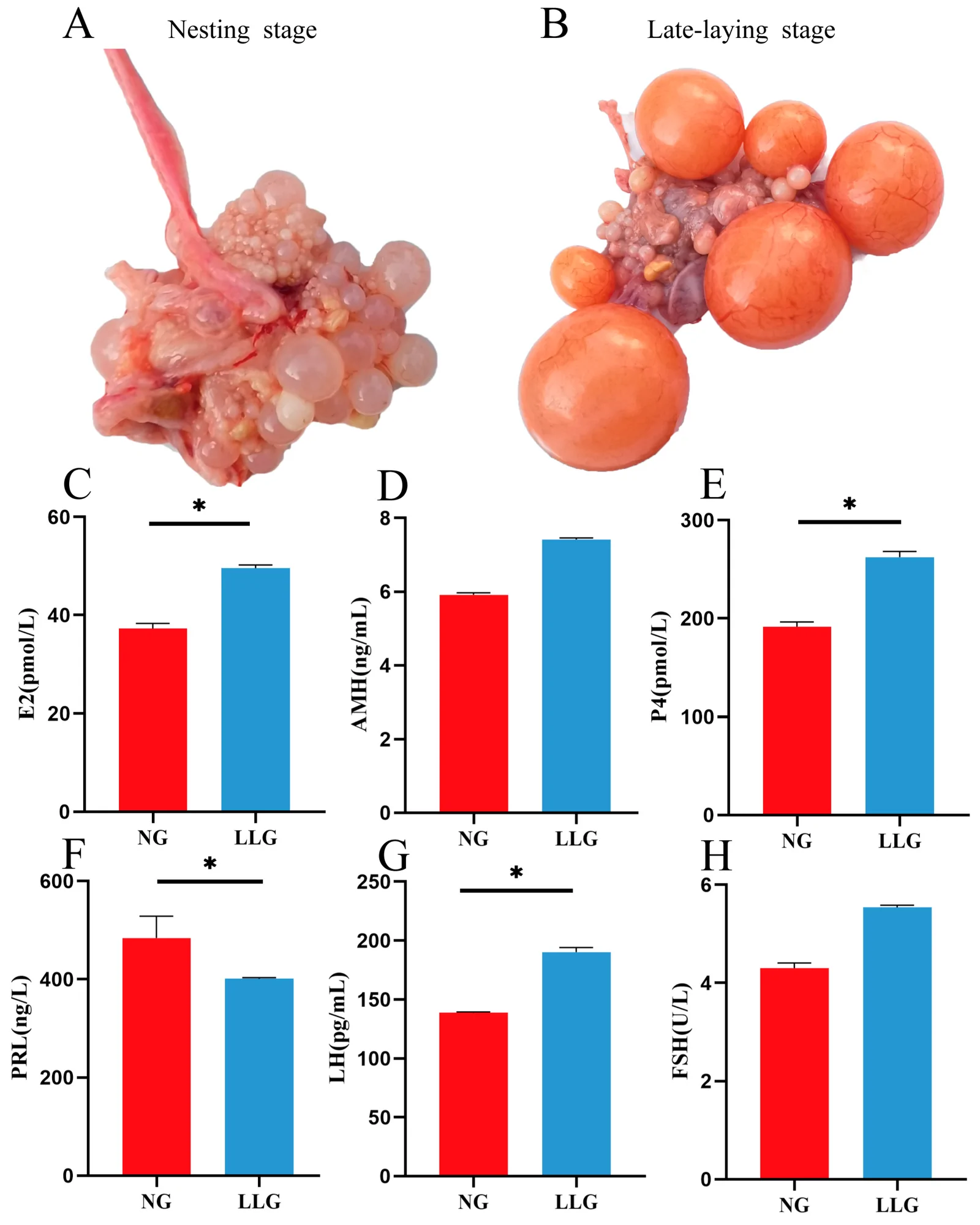
Morphological and hormonal changes during ovarian functional transition in Wanxi white geese. (**A**,**B**). Gross ovarian morphology of nesting (left) and late-laying (right) geese. (**C**–**H**). Serum concentrations of E2, AMH, P4, PRL, LH and FSH measured by ELISA. Data are presented as the mean ± s.d. (*n* = 10 birds per stage). * indicates statistically significant differences across the two reproductive stages (*p* < 0.05).

We evaluated ovarian structural changes and quantified the serum concentrations of reproductive hormones as geese progressed from late-laying into nesting (Figure 1). At the late-laying stage, the ovaries contained numerous large, well-developed preovulatory hierarchical follicles (Figure 1B). By contrast, ovaries of nesting geese showed marked regression and atrophy (Figure 1A). Mature hierarchical follicles were absent, and only a few small white and small yellow follicles remained. Serum hormone profiles were consistent with this morphological transition (Figure 1C–H). Compared with nesting geese, late-laying geese had significantly higher serum concentrations of E2, P4 and LH and a significantly lower concentration of PRL (all *p* < 0.05), whereas AMH and FSH did not differ significantly between the two stages (both *p* ≥ 0.05).

### 3.2. Quality Assessment of Ovarian circRNA and miRNA Sequencing Data

To assess sequencing quality and data yield, we analysed rRNA-depleted transcriptome and small RNA sequencing data from the LLG and NG groups. The six transcriptome libraries, which were used for circRNA identification, generated 567,948,958 raw reads. After adapter-containing and low-quality reads were removed, 561,974,986 clean reads were retained. Across the libraries, clean reads accounted for 98.23–99.15% of the total, while Q30 rates were 90.20–92.84%. Overall mapping rates, expressed relative to the reads remaining after rRNA removal, ranged from 50.64% to 72.41%, and GC content ranged from 54.12% to 62.37% (Table S2). Mapping efficiency was lowest in sample NG-1 (50.64%), which also showed the highest GC content (62.37%) and the lowest Q30 value (90.20%); this library nevertheless clustered with the remaining nesting replicates in downstream analyses and was therefore retained.

Small RNA sequencing yielded mean raw read counts of 12,869,589 and 13,552,587 per sample in the NG and LLG groups, respectively. High-quality clean reads accounted for 98.87% and 98.98% of the corresponding datasets. After reads lacking adapters or inserts and low-quality reads were removed, mean clean-tag counts of 12,552,255 and 13,287,161 per sample were retained in the NG and LLG groups, corresponding to 97.53% and 98.04% of the raw reads, respectively (Table 1).

### 3.3. RNA-Seq Profiling of Ovarian circRNAs at Late-Laying and Nesting Stages in Wanxi White Geese

**Figure 2:**
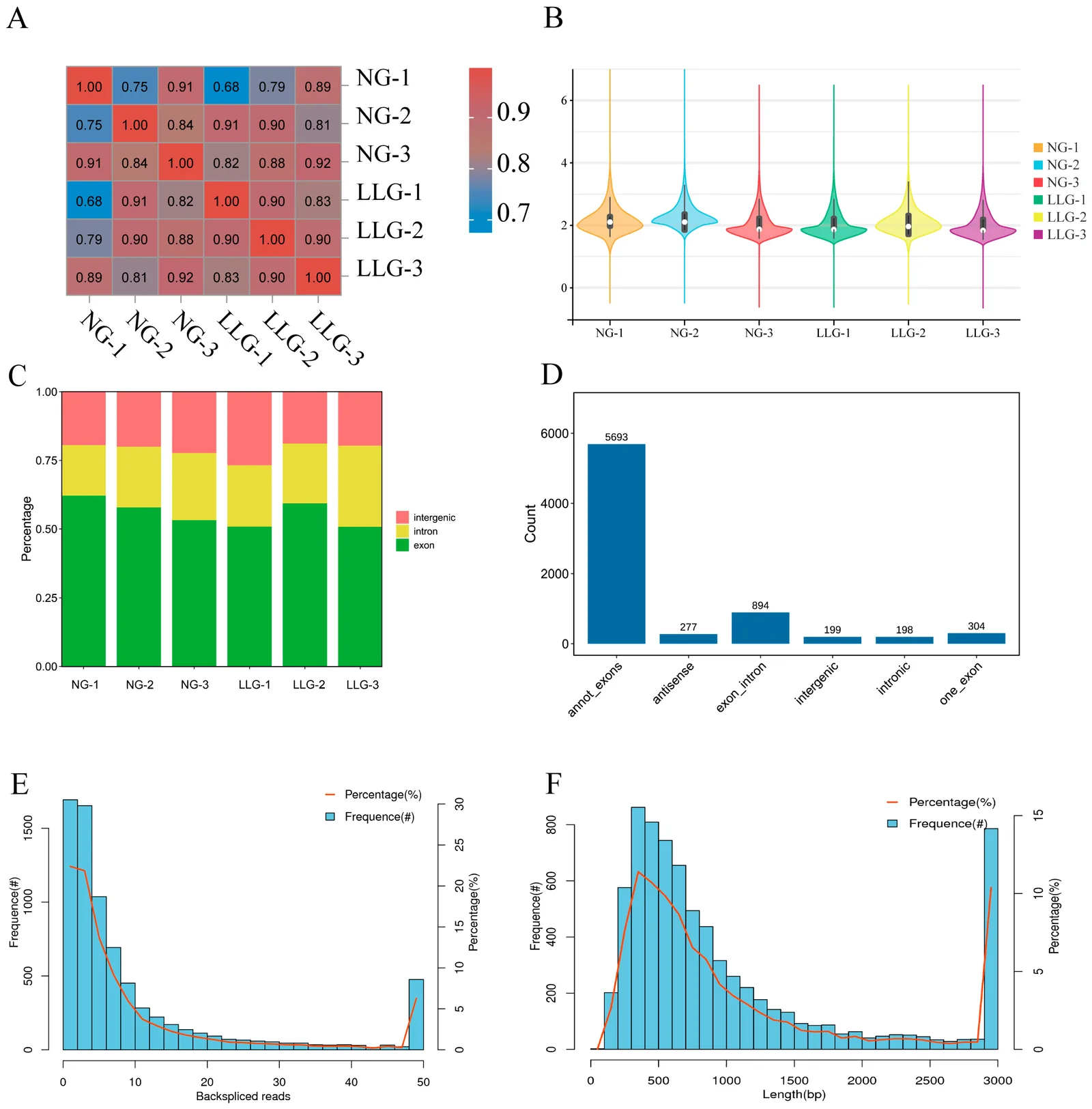
Expression and structural characteristics of circRNAs in Wanxi white goose ovarian tissue. (**A**). Pairwise Pearson correlations derived from circRNA abundance profiles. (**B**). Overall distribution of circRNA abundance within individual samples. (**C**). Proportions of circRNAs derived from different genomic regions. (**D**). Categories and numbers of identified circRNAs. (**E**). Distribution of BSJ-supporting read counts. (**F**). CircRNA length distribution. In panels (**E**,**F**), the symbol “#” denotes the number of circRNAs within each interval.

Pairwise Pearson correlation coefficients calculated from circRNA abundance profiles varied between 0.68 and 0.92, reflecting moderate-to-high concordance among samples (Figure 2A). The within-group coefficients ranged from 0.83 to 0.90 for LLG and from 0.75 to 0.91 for NG, indicating generally consistent circRNA expression among biological replicates; the lowest within-group coefficient (0.75, NG) may reflect biological or technical variation or sample-specific effects. The violin plots showed similar median expression levels and overall distributions across samples, with no obvious global shift between the LLG and NG groups (Figure 2B).

The sample-level distribution of circRNAs across genomic regions was broadly similar among samples. In every sample, exon-derived circRNAs predominated, whereas intronic and intergenic circRNAs each accounted for a small minority (Figure 2C). Structural classification of the combined dataset identified 7565 circRNAs, including 5693 annotated-exon (annot_exon; 75.3%), 894 exon-intron (11.8%), 304 single-exon (one_exon; 4.0%), 277 antisense (3.7%), 199 intergenic (2.6%) and 198 intronic (2.6%) circRNAs (Figure 2D). Annotated-exon circRNAs were thus by far the most abundant category, and exon-derived circRNAs (annot_exon, exon-intron and one_exon combined) accounted for 91.1% of the total.

Most circRNAs were supported by fewer than ten back-splice junction (BSJ)-spanning reads, whereas only a small proportion had higher read support (Figure 2E), consistent with the generally low abundance of circRNAs in ovarian tissue. CircRNA lengths were predominantly distributed between 200 and 1500 bp, with the largest number occurring between 300 and 500 bp. CircRNA frequency gradually declined with increasing length, although a subset of longer circRNAs was also detected (Figure 2F).

### 3.4. Identification of DE circRNAs in Wanxi White Goose Ovaries

**Figure 3:**
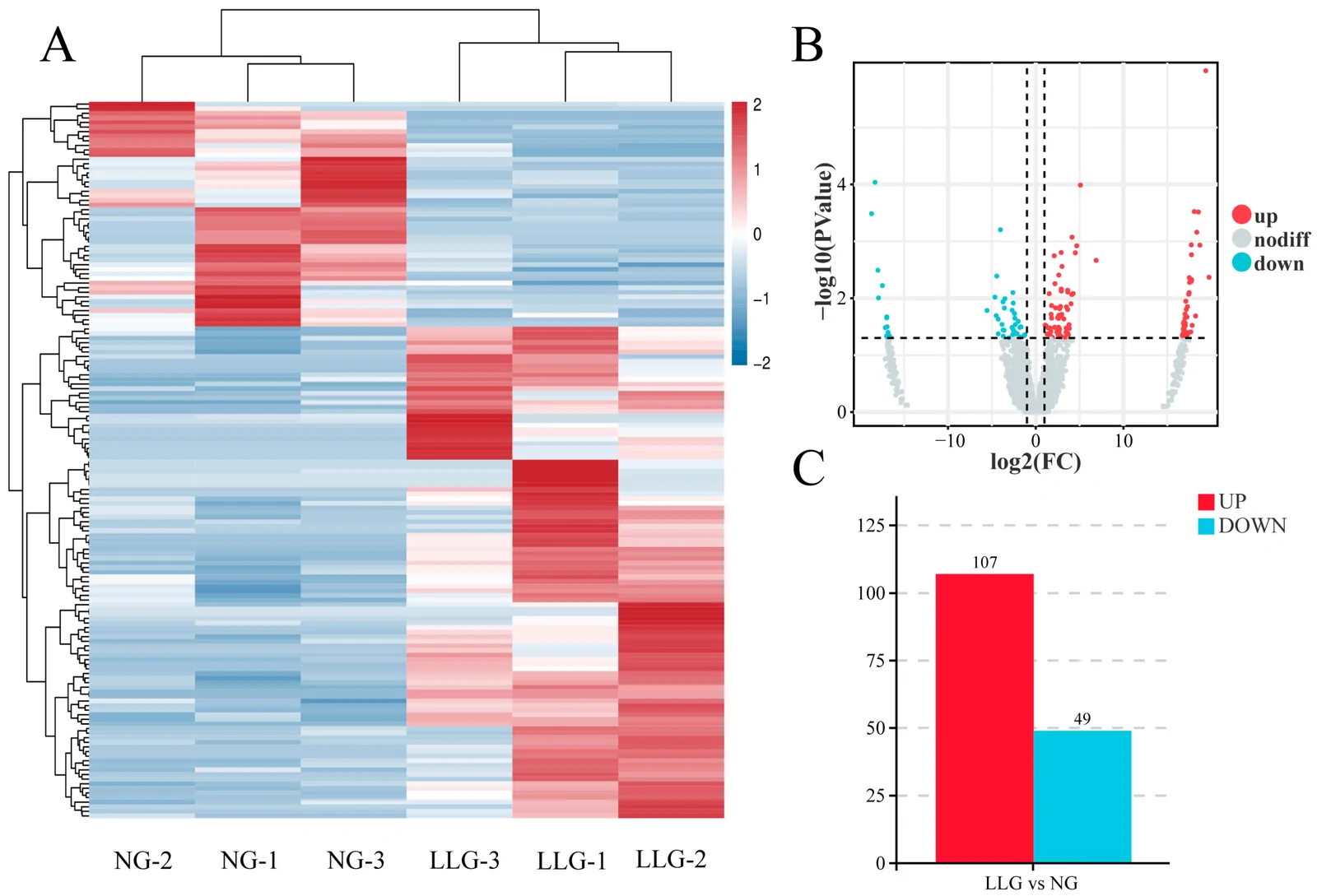
Stage-dependent circRNA abundance in Wanxi white goose ovaries across the shift from late laying to nesting. (**A**). Heatmap showing hierarchical clustering of DE circRNA abundance profiles. (**B**). Volcano plot summarising stage-associated circRNA changes. (**C**). Counts of circRNAs showing significantly higher or lower abundance in LLG than in NG.

We assessed the identified circRNAs for differential expression to define ovarian abundance changes from late-laying to nesting. Hierarchical clustering separated the NG and LLG samples into two distinct clusters, with biological replicates from the same group clustering closely together (Figure 3A). This result suggested stage-associated differences in the overall ovarian circRNA expression profiles.

Application of the |log_2_(fold change)| ≥ 1 and *p* < 0.05 criteria yielded 156 DE circRNAs. Of these, 107 were more abundant and 49 less abundant in LLG than in NG (Figure 3B,C, Supplementary Tables S4 and S5). Notably, novel_circ_000178, novel_circ_003577, novel_circ_005385, novel_circ_000559, novel_circ_000230, novel_circ_001280, novel_circ_000345 and novel_circ_000095 were more highly expressed, whereas novel_circ_002435, novel_circ_007511 and novel_circ_001139 were expressed at lower levels in LLG.

### 3.5. GO Terms and KEGG Pathways Enriched Among DE circRNA Host Genes

**Figure 4:**
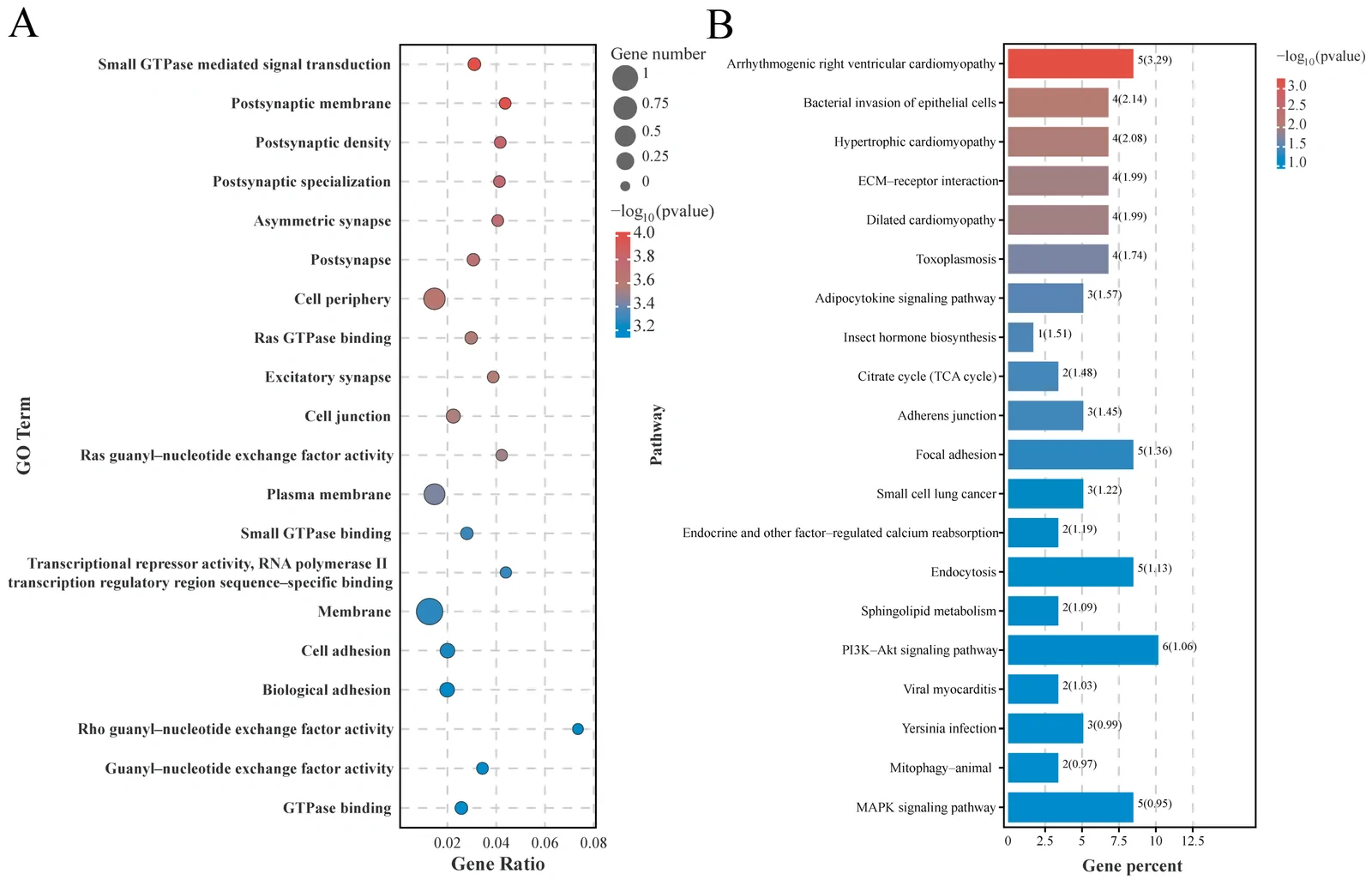
Functional enrichment profiles of genes hosting DE circRNAs. (**A**). Enriched GO terms for the host-gene set. (**B**). KEGG pathways enriched among the corresponding host genes.

We evaluated GO term and KEGG pathway enrichment among DE circRNA host genes to examine the possible involvement of these circRNAs in ovarian functional remodelling. Figure 4A revealed predominant enrichment of GO terms linked to membrane structures, cell junctions, and regulatory processes mediated by small GTPases. For cellular components, GO annotations enriched among the host genes included the cell junction, plasma membrane, and membrane. At the biological-process level, enrichment involved small GTPase-mediated signal transduction, cell adhesion, and biological adhesion. At the molecular-function level, enrichment involved GTPase binding, small GTPase binding, Ras GTPase binding, and Ras, Rho and general guanyl-nucleotide exchange factor activities. Collectively, these enrichment patterns linked DE circRNA host genes to membrane and junctional structures, small GTPase-mediated signal transduction, and cell adhesion.

KEGG analysis identified ECM–receptor interaction, adherens junction, focal adhesion, adipocytokine signalling and the citrate cycle (TCA cycle) as significantly enriched pathways among DE circRNA host genes (Figure 4B). ECM–receptor interaction, adherens junction and focal adhesion are associated with tissue structural remodelling. The adipocytokine signalling pathway and citrate cycle are associated with metabolic regulation. These findings suggest that DE circRNAs may participate in ovarian functional transition through processes related to tissue structural remodelling and metabolic regulation.

### 3.6. Ovarian Expression Patterns of DE miRNAs in Wanxi White Geese at the Nesting and Late-Laying Stages

Across the six samples, 496 known miRNAs were identified, with 73,913,885 tags assigned to these miRNAs, accounting for 95.35% of all small RNA tags. Individual samples contained 391–439 known miRNAs, and tags assigned to known miRNAs accounted for 93.83–97.27% of all tags in each sample. In addition, 125 candidate novel miRNAs were predicted, with 25,436 assigned tags, accounting for 0.03% of all tags. Individual samples contained 85–107 candidate novel miRNAs, and tags assigned to these candidates accounted for 0.02–0.04% of all tags (Table 2).

**Figure 5:**
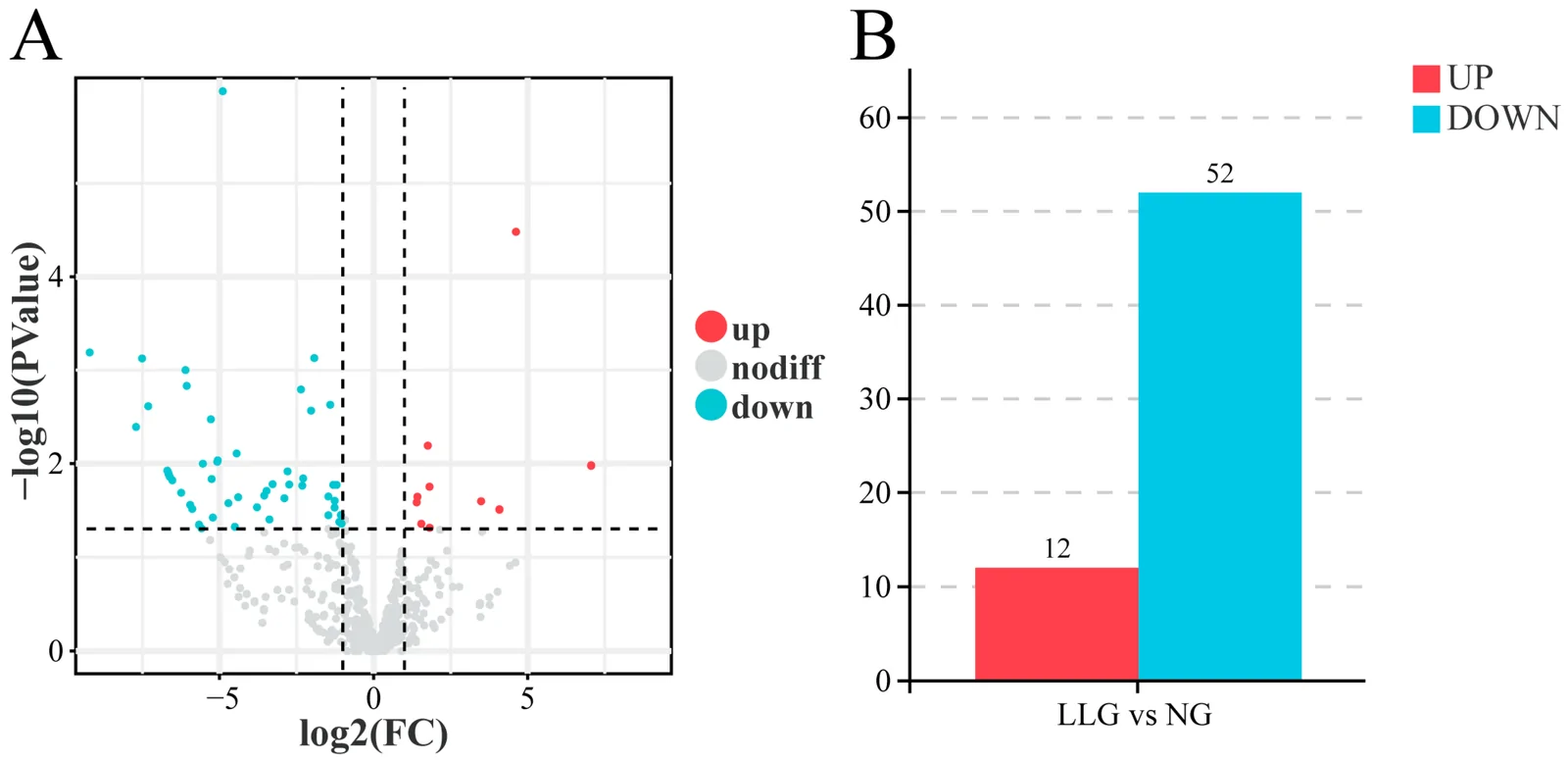
Differential ovarian miRNA profiles across the nesting and late-laying stages of Wanxi white geese. (**A**). Volcano plot illustrating DE miRNAs in the comparison between NG and LLG. (**B**). Counts of miRNAs whose expression was significantly higher or lower in LLG than in NG.

Comparative analysis of ovarian miRNA expression profiles between the NG and LLG periods identified 64 DE miRNAs (Figure 5A, Supplementary Tables S4 and S6). Relative to NG, 12 miRNAs were significantly upregulated and 52 were significantly downregulated in LLG (Figure 5B); that is, miRNA expression was predominantly higher in nesting ovaries. Within the DE miRNA set, miR-1-y, miR-25-y, miR-144-y, miR-184-y, miR-185-x, miR-187-x, miR-215-y, miR-223-y, miR-342-y, miR-365-y, miR-490-y, miR-1805-x, and miR-4029-x showed significant downregulation at the late-laying stage. In contrast, miR-31-x, miR-72-x, miR-135-x, miR-135-z and novel-m0009-3p were significantly upregulated during the late-laying period.

### 3.7. GO Term and KEGG Pathway Enrichment Among Predicted Gene Targets of DE miRNAs

**Figure 6:**
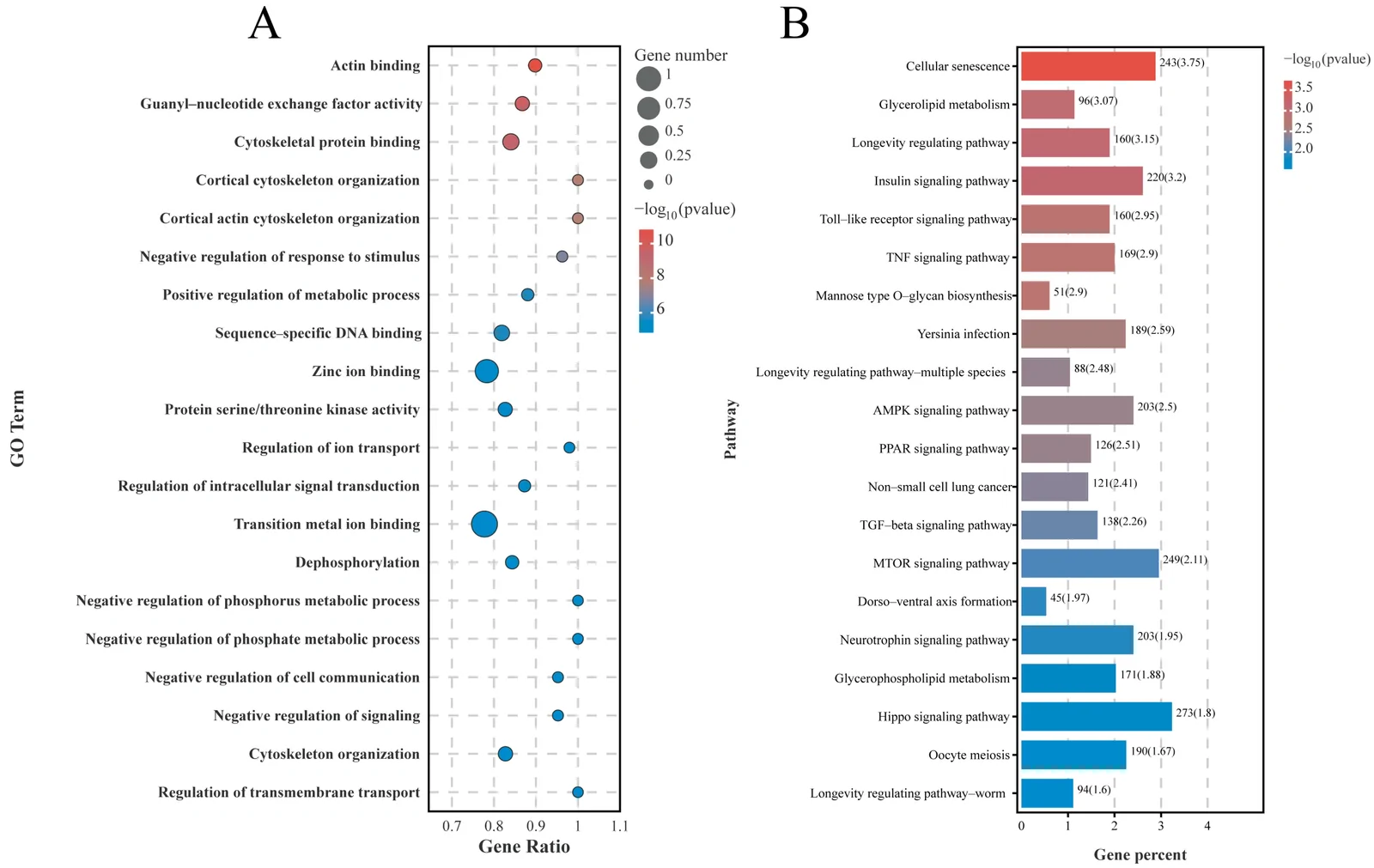
Functional enrichment profiles of genes predicted as targets of DE miRNAs in Wanxi white goose ovaries at the nesting and late-laying stages. (**A**). GO terms enriched in the predicted target set. (**B**). KEGG pathways enriched among the corresponding genes.

To explore the possible functional roles of DE miRNAs, we assessed GO term and KEGG pathway enrichment among their predicted gene targets. GO enrichment in Figure 6A centred on cytoskeletal organisation, signal transduction, and regulation of protein phosphorylation. At the biological-process level, enrichment involved cortical actin cytoskeleton organisation and cytoskeleton organisation. Additional terms included protein dephosphorylation and regulation of intracellular signal transduction. At the molecular-function level, enriched terms comprised protein serine/threonine kinase activity and actin binding. Overall, the enrichment profile indicated associations between predicted DE miRNA target genes and cytoskeletal remodelling, intracellular signalling, and protein phosphorylation.

KEGG analysis identified prominent pathway enrichments among genes predicted to be targeted by DE miRNAs. These comprised cellular senescence, oocyte meiosis, and the AMPK, TGF-β, mTOR, and Hippo signalling pathways (Figure 6B). Enrichment in cellular senescence was associated with ovarian cell senescence, apoptosis and follicular atresia, whereas enrichment in oocyte meiosis was related to oocyte maturation and ovulation. The AMPK and mTOR signalling pathways were associated with energy sensing and metabolic remodelling. Moreover, the TGF-β and Hippo signalling pathways were associated with the proliferation and differentiation of follicular cells, as well as tissue remodelling.

### 3.8. Assembly of Candidate ceRNA Networks Linking circRNAs, miRNAs and mRNAs

**Figure 7:**
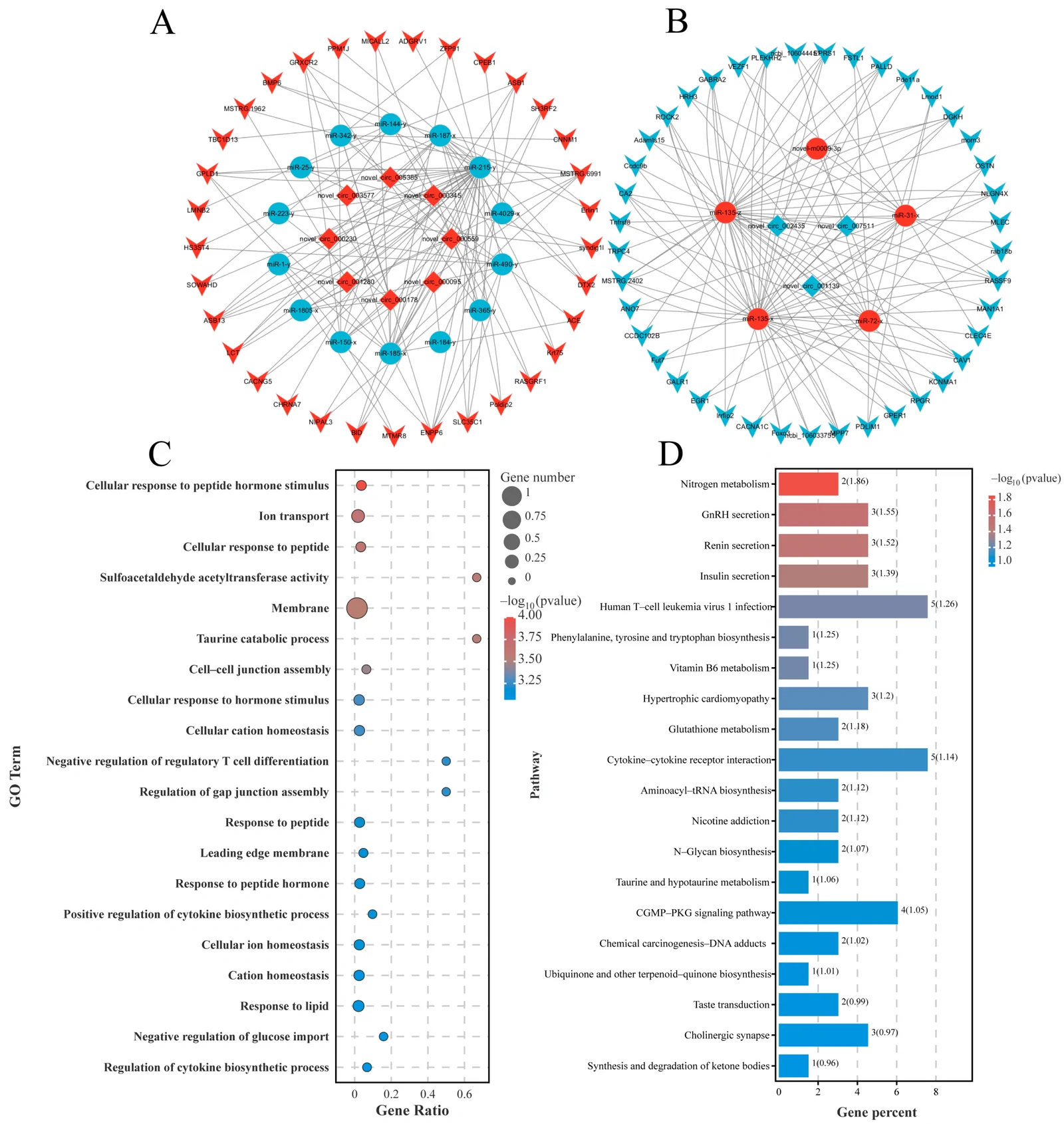
Candidate circRNA–miRNA–mRNA networks and functional annotation of target mRNAs in Wanxi white goose ovaries at the nesting and late-laying stages. (**A**). Network of circRNAs and mRNAs showing higher expression in LLG and miRNAs showing lower expression, with NG as the reference. (**B**). Network of circRNAs and mRNAs showing lower expression in LLG and miRNAs showing higher expression, with NG as the reference. Circles denote miRNAs, V-shaped nodes denote mRNAs, and diamonds denote circRNAs. Predicted regulatory interactions are shown as grey edges. Red and blue nodes mark significant upregulation and downregulation, respectively, in LLG compared with NG. (**C**). GO terms enriched among target mRNAs from the candidate ceRNA networks. (**D**). KEGG pathways enriched within the same mRNA set.

We assembled two exploratory regulatory networks linking circRNAs, miRNAs and mRNAs that met the nominal DE criteria to examine their possible interactions during ovarian functional remodelling in Wanxi white geese. Network assembly incorporated expression changes observed in LLG compared with NG (Figure 7A,B, Supplementary Table S8).

In the network shown in Figure 7A, eight circRNAs were upregulated during the late-laying period. These included novel_circ_000178, novel_circ_003577, novel_circ_005385, novel_circ_000559, novel_circ_000230, novel_circ_001280, novel_circ_000345 and novel_circ_000095. By contrast, their potentially interacting miRNAs were downregulated, whereas their predicted target mRNAs were predominantly upregulated. These miRNAs included miR-1-y, miR-25-y, miR-144-y, miR-1805-x, miR-184-y, miR-185-x, miR-187-x, miR-215-y, miR-223-y, miR-342-y, miR-365-y, miR-490-y and miR-4029-x. This pattern was consistent with greater transcript abundance in LLG among genes potentially linked to follicular development and the maintenance of laying activity.

Conversely, novel_circ_002435, novel_circ_007511 and novel_circ_001139 were downregulated during the late-laying period in the network shown in Figure 7B. MiR-31-x, miR-72-x, miR-135-x, miR-135-z and novel-m0009-3p were upregulated, whereas their predicted target mRNAs were predominantly downregulated. These mRNAs were therefore expressed at higher levels during the nesting period and may be associated with ovarian regression, endocrine changes and tissue remodelling.

Across both networks, circRNAs and mRNAs exhibited concordant expression patterns, whereas miRNAs showed the opposite pattern. These expression patterns were consistent with the predicted ceRNA relationships, which require functional validation.

We characterised the candidate ceRNA networks by assessing functional enrichment among their constituent target genes. The leading GO terms involved membrane-associated functions, cellular responses to hormone stimuli, ion transport, cell–cell junction assembly, cellular ion homeostasis and cation homeostasis (Figure 7C). The significantly enriched KEGG pathways included nitrogen metabolism, GnRH secretion, renin secretion and insulin secretion (Figure 7D). These functional profiles suggested potential involvement of the candidate ceRNA networks in endocrine signalling, ion homeostasis and metabolic regulation during ovarian functional transition.

To provide a concise overview of key network components, 12 mRNAs showing network-consistent expression patterns were summarised in Supplementary Tables S3, S4 and S7. CPEB1, BMP6, LPIN3 and PIP5K1C were more highly expressed during the late-laying period. By contrast, FOXO3, CACNA1C, FSTL1, HRH3, IQSEC3, KCNQ5, NCALD and GABRA2 were more highly expressed during the nesting period. Their gene-level annotations involved oocyte maturation, ovarian steroidogenesis, hormone and calcium signalling, lipid metabolism and neuroendocrine regulation. Specifically, CPEB1 and BMP6 were annotated to oocyte maturation and ovarian steroidogenesis, respectively. CACNA1C was associated with GnRH secretion and calcium signalling, whereas FOXO3, HRH3, KCNQ5 and GABRA2 were linked to FoxO, neuroactive receptor or synaptic signalling pathways. Together, the network expression patterns and functional annotations supported putative relationships associated with the transition from late laying to nesting.

### 3.9. Identification of Candidate ceRNA Relationships Associated with the Late-Laying and Nesting Stages

**Figure 8:**
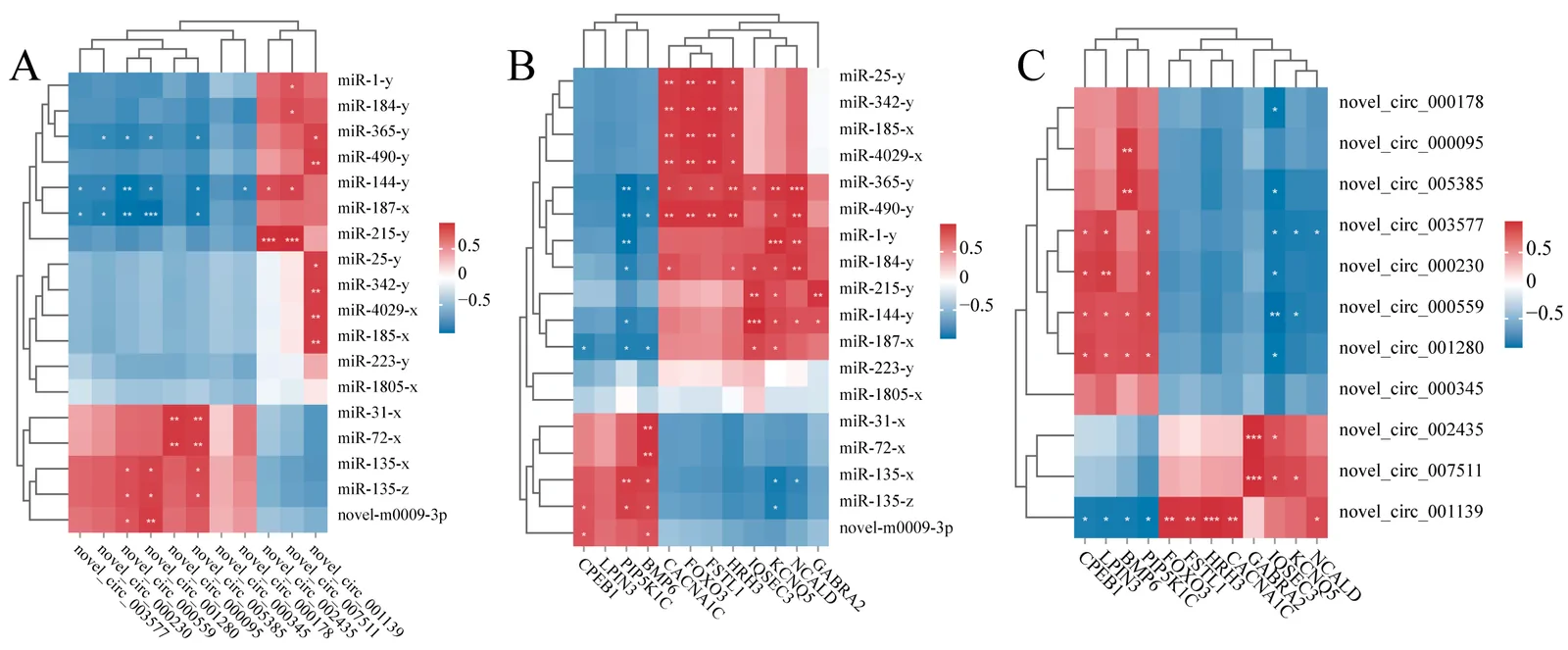
Pearson correlations among the expression profiles of circRNAs, miRNAs and mRNAs included in candidate ceRNA networks. (**A**). Heatmap depicting expression correlations between candidate circRNAs and miRNAs. (**B**). Expression correlations linking candidate miRNAs to their target mRNAs, displayed as a heatmap. (**C**). Correlation heatmap between candidate circRNAs and target mRNAs. Pearson correlations were calculated across six biological samples (three per stage). * *p* < 0.05, ** *p* < 0.01 and *** *p* < 0.001.

Expression-pattern concordance within the candidate ceRNA networks was examined through Pearson correlations derived from the expression profiles of selected circRNAs, miRNAs and mRNAs (Figure 8). The circRNA–miRNA correlation analysis revealed two relatively distinct modules with opposing expression patterns (Figure 8A). novel_circ_000178, novel_circ_000095, novel_circ_005385, novel_circ_003577, novel_circ_000230, novel_circ_000559, novel_circ_001280 and novel_circ_000345, which were upregulated during the late-laying period, were generally negatively correlated with miR-1-y, miR-184-y, miR-365-y, miR-490-y, miR-144-y, miR-187-x and miR-215-y, which were upregulated during the nesting period. Conversely, novel_circ_002435, novel_circ_007511 and novel_circ_001139, which were downregulated during the late-laying period, were negatively correlated with miR-31-x, miR-72-x, miR-135-x, miR-135-z and novel-m0009-3p, which were downregulated during the nesting period. These correlation patterns were compatible with the candidate circRNA–miRNA relationships predicted under the ceRNA model.

The miRNA–mRNA correlation analysis also revealed distinct modular patterns (Figure 8B). Expression of miR-1-y, miR-184-y, miR-187-x and miR-215-y was negatively correlated with the laying-associated candidate genes CPEB1, LPIN3, PIP5K1C and BMP6. The expression levels of miR-31-x, miR-72-x, miR-135-x, miR-135-z and novel-m0009-3p were negatively correlated with candidate genes including CACNA1C, FOXO3, FSTL1, HRH3, IQSEC3, KCNQ5, NCALD and GABRA2. These negative correlations were consistent with the predicted miRNA–mRNA relationships and warrant further experimental evaluation.

The circRNA–mRNA correlation analysis further revealed positive co-expression patterns (Figure 8C). The eight circRNAs upregulated during the late-laying period were positively correlated with CPEB1, LPIN3, PIP5K1C and BMP6, which were also upregulated during this period. Conversely, novel_circ_002435, novel_circ_007511 and novel_circ_001139 showed positive correlations with candidate genes, including FOXO3 and FSTL1. These genes exhibited lower expression in LLG and higher expression in NG. Together, these correlation patterns, combined with target predictions and differential expression profiles, were used to define candidate ceRNA relationships associated with ovarian functional transition in Wanxi white geese.

### 3.10. Correlations Between Serum Hormone Concentrations and Expression of Candidate ceRNA Components

**Figure 9:**
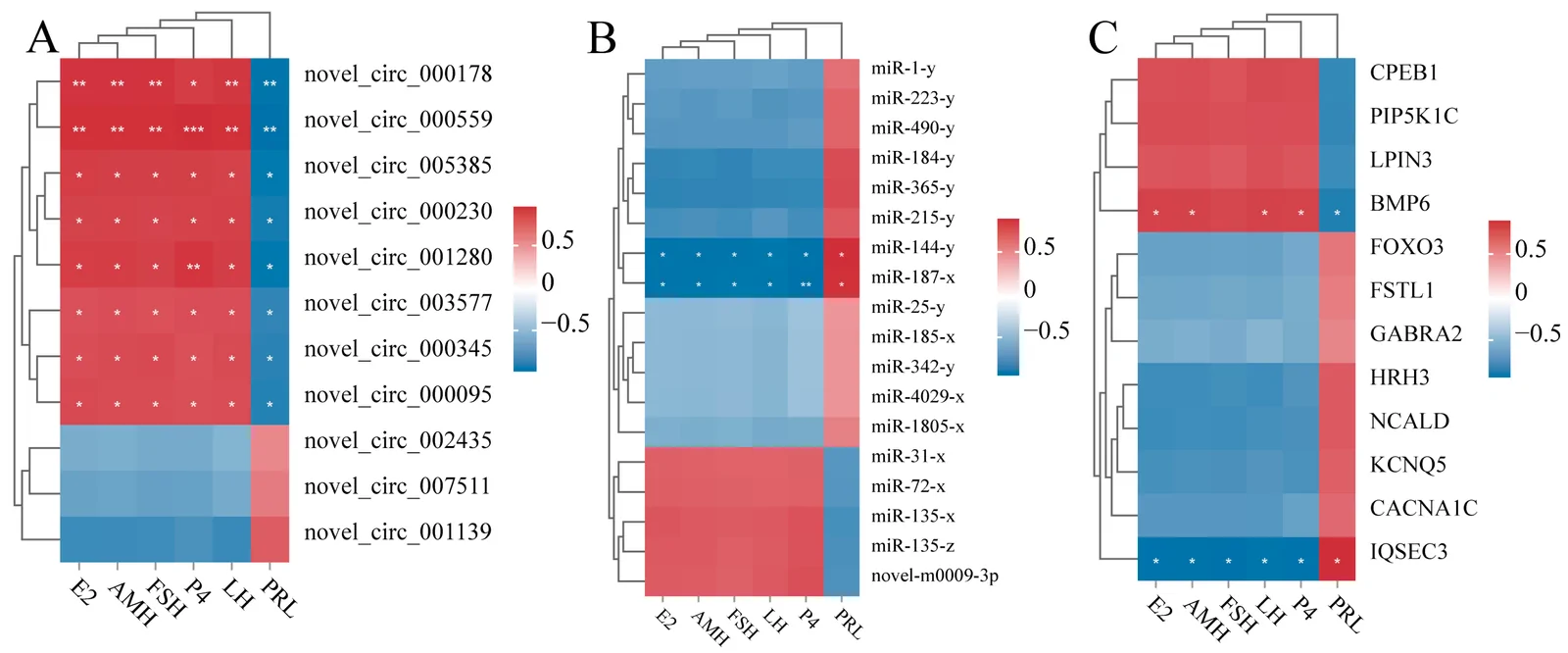
Pearson correlations linking the expression profiles of circRNAs, miRNAs and mRNAs from the candidate ceRNA networks to serum reproductive hormone levels. (**A**). Heatmap displaying correlations of candidate circRNAs with six serum hormones including E2, AMH, P4, PRL, FSH and LH. (**B**). Heatmap illustrating associations of candidate miRNAs with the same hormone panel. (**C**). Corresponding correlation map for candidate mRNAs and the six hormones. Pearson correlations were calculated across six biological samples (three per stage). * *p* < 0.05, ** *p* < 0.01 and *** *p* < 0.001.

Potential associations between the candidate ceRNA regulatory networks and reproductive endocrine phenotypes were further examined in Wanxi white geese. Pearson correlation coefficients linking the expression profiles of selected DE circRNAs, miRNAs and mRNAs to serum E2, AMH, P4, PRL, FSH and LH concentrations were subsequently calculated (Figure 9).

The circRNA–hormone correlation analysis showed that most candidate circRNAs, including novel_circ_003577, novel_circ_000345, novel_circ_000095 and novel_circ_005385, were positively correlated with E2, AMH and P4, as well as FSH and LH, but negatively correlated with PRL (Figure 9A). By contrast, novel_circ_002435, novel_circ_007511 and novel_circ_001139 correlated positively with PRL but negatively with E2, AMH, P4, FSH and LH.

The miRNA–hormone correlation analysis showed that miR-1-y, miR-223-y, miR-490-y, miR-184-y and miR-365-y were negatively correlated with E2, AMH and P4, as well as FSH and LH, but positively correlated with PRL (Figure 9B). Expression of miR-31-x, miR-72-x, miR-135-x and novel-m0009-3p displayed the opposite correlation pattern.

For mRNAs, CPEB1, BMP6, PIP5K1C and LPIN3 were positively correlated with E2, AMH and P4, as well as FSH and LH, but negatively correlated with PRL (Figure 9C). Conversely, positive correlations with PRL were observed for FOXO3, FSTL1, GABRA2, HRH3, NCALD, KCNQ5, CACNA1C and IQSEC3, whereas their associations with E2, AMH, P4, FSH and LH were negative. These findings showed stage-associated correlations between reproductive hormone concentrations and the expression of candidate ceRNA components.

### 3.11. RT-qPCR Assessment of Expression Patterns for mRNAs, circRNAs and miRNAs

**Figure 10:**
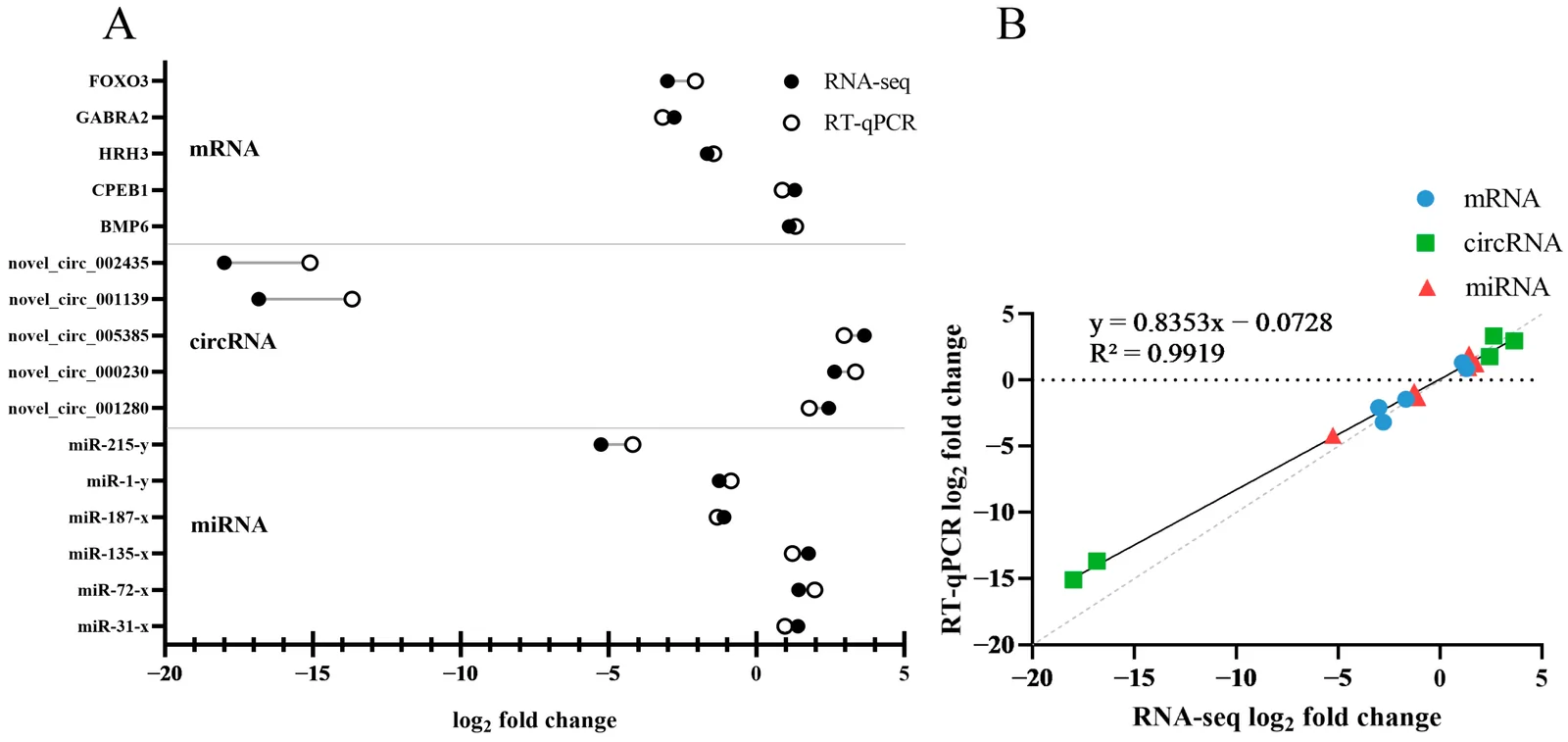
RT-qPCR verification of differential expression in selected mRNAs, miRNAs and circRNAs. (**A**). Comparison of log_2_ fold changes determined by RNA-seq and RT-qPCR for 16 candidate RNAs in LLG relative to NG. Filled and open circles represent RNA-seq and RT-qPCR results, respectively. Grey lines connect measurements for the same RNA, and the vertical dashed line indicates log_2_ fold change = 0. (**B**). Scatter plot comparing RNA-seq-derived measurements with RT-qPCR measurements. The grey dashed diagonal marks the line of identity (y = x), whereas the fitted linear regression is shown by a solid black line. RNA classes are distinguished by symbol: blue circles for mRNAs, green squares for circRNAs and red triangles for miRNAs.

RT-qPCR was used to quantify selected DE mRNAs, DE circRNAs and DE miRNAs, allowing the expression patterns derived from RNA-seq to be assessed using a second method in the same biological samples. The RT-qPCR measurements generally reproduced the trends identified by RNA-seq, and the selected RNAs changed in the same direction when assessed by either method (Figure 10). Correlation analysis further showed concordance between the RT-qPCR and RNA-seq measurements for the selected RNAs. Linear regression of the RT-qPCR against the RNA-seq log_2_ fold changes for the 16 selected RNAs yielded R^2^ = 0.9919, supporting quantitative concordance between the two platforms.

## 4. Discussion

The transition of the avian ovary from an active laying state to a functionally quiescent nesting state is characterised not only by reduced follicle numbers and the cessation of ovulation, but also by coordinated changes in hypothalamic–pituitary–ovarian axis function, local ovarian endocrine activity and cellular state [3]. This transition was reflected in both morphological and hormonal findings of the present study. During the late-laying period, the ovaries contained numerous well-developed hierarchical preovulatory follicles. Serum E2 and P4 concentrations remained relatively high [17,18], suggesting that follicular development, steroidogenesis and ovulatory activity were still maintained. Upon entry into the nesting period, mature hierarchical follicles disappeared and the ovaries underwent marked regression [19]. This was accompanied by decreases in E2, P4 and LH concentrations and a significant increase in PRL [20,21], whereas AMH and FSH did not differ significantly between the two stages. The reductions in E2 and P4 may reflect diminished steroidogenic activity in granulosa and theca cells. The decrease in LH may reflect reduced pituitary gonadotropic support, potentially limiting follicular growth, steroidogenesis and ovulation. The increase in PRL is consistent with the maintenance of nesting behaviour and suppression of the reproductive axis. Together, these changes are consistent with ovarian regression during nesting involving coordinated shifts in reproductive programmes. Programmes supporting follicular growth and ovulation may differ from those associated with cellular quiescence, stress adaptation and tissue remodelling.

CircRNAs are increasingly recognised as important post-transcriptional regulators of animal reproduction because of their high stability and pronounced tissue and developmental-stage specificity [22]. Analysis of the ovarian circRNA transcriptome in Wanxi white geese showed that most of the identified circRNAs (approximately 75%) originated from the exonic regions of known protein-coding genes and were therefore classified as exonic circRNAs. This genomic distribution was broadly consistent with previous findings in humans [23], mice [24] and chickens [25]. The distribution of circRNA types observed in the present study was broadly consistent with previous ovarian circRNA expression profiles reported in geese [14]. Exonic circRNAs can be transported to the cytoplasm, where they exert their biological functions [26]. This spatial distribution provides a structural and biological basis for further investigating whether these molecules may act as miRNA sponges through the ceRNA mechanism during ovarian functional transition [27]. Notably, neither the overall distribution of circRNA expression nor the composition of circRNA types changed markedly between the late-laying and nesting ovaries of Wanxi white geese, although several circRNAs showed stage-associated differential expression under the nominal screening criteria. This observation suggests that ovarian functional transition may not result from a general increase or decrease in overall circRNA production, but may instead involve the selective remodelling of specific circRNAs and their downstream regulatory relationships.

One important mechanism through which circRNAs participate in post-transcriptional regulation is competitive binding to miRNAs, thereby modulating miRNA-mediated repression of downstream target mRNAs. Therefore, further elucidation of the roles of DE circRNAs in ovarian functional transition requires an integrated analysis of changes in miRNA expression, target-gene functions and the expression relationships among circRNAs, miRNAs and mRNAs. In this study, we integrated circRNA, miRNA and mRNA expression data to construct a potential exploratory ceRNA network associated with ovarian functional transition in Wanxi white geese. The results suggest that the transition from the late-laying period to the nesting period may involve coordinated changes in multiple post-transcriptional regulatory modules. These putative modules may be associated with oocyte maturation, granulosa-cell function, energy metabolism and follicular atresia, rather than the transition being determined by a single gene or signalling pathway.

In chicken granulosa cells, miR-223 directly targets CRIM1, suppresses AKT signalling activity, and reduces granulosa-cell proliferation and steroidogenic capacity [28]. MiR-223 overexpression also alters the abundance of STAR, CYP11A1 and other genes involved in steroidogenesis. In the present study, miR-223-y was upregulated during the nesting period, when mature hierarchical follicles disappeared and serum E2 and P4 concentrations declined. Therefore, increased miR-223-y expression may be associated with stronger repression of targets involved in granulosa-cell proliferation and steroidogenesis; whether this contributes to arrested follicular development or reduced ovarian endocrine function remains to be tested.

In mouse granulosa cells, miR-144 can target COX-2 and reduce prostaglandin E2 production [29]. COX-2 and prostaglandin signalling participate in preovulatory follicular responses, follicular-wall remodelling and oocyte release. Increased miR-144 expression may therefore weaken the follicular response to ovulatory stimuli. In the present study, miR-144-y was upregulated during the nesting period. However, miR-144 is highly expressed in healthy bovine large follicles but decreases in atretic follicles. This suggests that its expression and function may be species-specific and jointly influenced by reproductive status, follicular development or atresia, and target-gene composition [30]. Therefore, the role of miR-144-y in Wanxi white geese cannot be simply defined as promoting or inhibiting follicular development, but may depend on the specific target mRNAs that it binds in the nesting ovary.

A previous study detected differential expression of miR-215-5p in goat large follicles, suggesting a possible association with follicular status [31]. In this study, miR-215-y displayed an inverse correlation with BMP6, a gene previously linked to laying performance. This association suggests that miR-215-y could contribute to the post-transcriptional control of granulosa-cell activity and signalling involved in follicle growth.

Among the miRNAs downregulated during the nesting period, miR-31-x is associated with ovarian responsiveness to gonadotropins. Studies have shown that miR-31 can target FSHR in bovine follicular granulosa cells and affect steroid hormone synthesis and cell survival [32]. FSHR is an important receptor through which granulosa cells respond to FSH stimulation and maintain follicular growth. Changes in miR-31 expression may therefore alter the sensitivity of follicular cells to gonadotropins. Although miR-31-x was downregulated during the nesting period in the present study, serum E2 and P4 concentrations still declined markedly, suggesting that reduced miR-31-x expression was not accompanied by restored follicular function. This change may represent a compensatory response following ovarian functional decline. Alternatively, miR-31-x may primarily affect target genes associated with tissue remodelling, ion channels and cell communication rather than directly restoring steroidogenesis.

The miR-135-x/z pair may provide a putative link among TGF-β signalling, granulosa-cell growth and FOXO3-related functions. miR-135a has been shown to target TGFBR1 and CCND2 in mouse granulosa cells, thereby inhibiting cell-cycle progression and cell proliferation. This finding indicates that miR-135 may participate in regulating granulosa-cell growth during folliculogenesis [33]. In the present study, miR-135-x/z was downregulated during the nesting period, whereas predicted targets such as FOXO3 were upregulated. FOXO3 is closely associated with follicular quiescence, cellular stress and follicular atresia. Therefore, downregulation of miR-135-x/z may reduce the post-transcriptional repression of FOXO3, which could alter FOXO3-related activity and may be associated with ovarian functional decline during the nesting period.

Although the miRNAs described above have been implicated in granulosa-cell proliferation, steroidogenesis, follicular maturation and atresia, individual miRNAs typically act on several gene targets. Conversely, the same target gene may be jointly regulated by multiple miRNAs and circRNAs. Therefore, changes in the expression of individual miRNAs alone cannot fully explain the complex molecular mechanisms underlying ovarian functional transition in Wanxi white geese. By integrating the expression directions of circRNAs, miRNAs and mRNAs with target predictions and correlation relationships, we further identified two putative ceRNA regulatory patterns with opposing expression directions. These opposing patterns suggest that the attenuation of laying-related programmes and the enhancement of broodiness-associated programmes of ovarian regression may occur simultaneously.

Based on these network features, we further identified several putative ceRNA regulatory axes, including novel_circ_001280-miR-1-y-CPEB1, novel_circ_005385-miR-187-x-BMP6 and novel_circ_002435/novel_circ_007511-miR-135-x/z-FOXO3. Among these, the novel_circ_001280-miR-1-y-CPEB1 axis may primarily contribute to maintaining maternal mRNA translational regulation and meiotic maturation in oocytes during the late-laying period. In late-laying ovaries, novel_circ_001280 was expressed at a relatively high level. This circRNA is derived from the NBEA (neurobeachin) gene, which encodes a scaffold protein associated with vesicular trafficking and membrane-protein localisation and is expressed in various secretory cell types [34]. Given the active membrane-receptor trafficking, signal transduction and steroidogenic activities of ovarian granulosa cells, NBEA-related vesicular trafficking may be associated with granulosa-cell endocrine function. However, the function of a host gene cannot be directly equated with that of its derived circRNA. Therefore, the biological function of NBEA mainly provides information about the possible genomic and functional background of novel_circ_001280, whereas the specific regulatory role of this circRNA should be interpreted within the ceRNA network.

Previous studies have shown that cholesterol transport, steroidogenic-enzyme localisation and secretion-related processes in granulosa cells are important for E2 and P4 synthesis and follicular maturation [35]. In the present study, novel_circ_001280 was negatively correlated with miR-1-y but positively correlated with CPEB1, whereas miR-1-y was negatively correlated with CPEB1. Following the transition to the nesting period, decreased novel_circ_001280 expression may reduce its predicted competitive binding to miR-1-y, potentially enhancing miR-1-y-mediated repression of CPEB1. CPEB1 is an important regulator of the cytoplasmic polyadenylation and translational activation of maternal mRNAs in oocytes. It also participates in oocyte growth, meiotic resumption and maturation [36]. Therefore, decreased CPEB1 expression may weaken the translational activation of stored mRNAs in oocytes, thereby affecting meiotic progression and maturation competence. The novel_circ_001280-miR-1-y-CPEB1 axis is therefore consistent with a preliminary module associated with maternal mRNA translational regulation and the oocyte-maturation programme during the late-laying period. Lower expression of these components may be associated with the regression of hierarchical follicles and the cessation of ovulation during the nesting period.

In addition to oocyte maturation, the maintenance of ovarian function during the late-laying period depends on granulosa-cell proliferation and differentiation, as well as local steroidogenic activity. Based on the ceRNA network analysis, we further identified BMP6 as a potential downstream node linking non-coding RNA regulation to granulosa-cell function. During the late-laying period, circRNAs including novel_circ_000095, novel_circ_000230, novel_circ_005385 and novel_circ_001280 were expressed at relatively high levels, whereas miRNAs including miR-184-y, miR-187-x and miR-215-y were expressed at relatively low levels. BMP6 also maintained a relatively high expression level. This pattern was consistent with a predicted ceRNA relationship. Among these interactions, novel_circ_005385-miR-187-x-BMP6 may represent a putative regulatory axis within the network.

In the present study, miR-187-x was more highly expressed in the ovary during the nesting period. Previous studies have shown that the miR-187 family can participate in the regulation of BMP/TGF-β-related signalling and affect the functional state of cumulus or granulosa cells [37]. BMP6 is a member of the TGF-β superfamily and can regulate granulosa-cell differentiation, FSH responsiveness, AMH expression and steroidogenesis through BMP receptors and SMAD1/5/8 signalling [38]. The decrease in novel_circ_005385 expression during the nesting period may reduce its predicted competitive binding to miR-187-x, potentially enhancing miR-187-x-mediated repression of BMP6. This change may subsequently affect BMP-BMPR-SMAD1/5/8 signalling and granulosa-cell function.

The effects of BMP6 on steroidogenesis may vary according to species, follicular stage and cellular context. Therefore, reduced BMP6 expression is more appropriately interpreted as a potential change in local granulosa-cell signalling and endocrine responsiveness rather than simply equated with a unidirectional decrease in oestrogen or progesterone synthesis. Our analysis of the predicted targets of DE miRNAs highlighted both the TGF-β and Hippo signalling pathways. Hippo signalling can respond to cell density, mechanical tension and changes in the extracellular matrix. It can also interact with TGF-β/BMP signalling to affect granulosa-cell proliferation and differentiation and the stability of the follicular microenvironment [39]. Therefore, the regulatory network comprising multiple circRNAs, miRNAs and BMP6 may integrate BMP signalling, cell proliferation and changes in follicular structure. By coordinating these processes, the network may help preserve follicular function in LLG while dampening programmes that promote follicle growth in NG [40]. Overall, the novel_circ_005385-miR-187-x-BMP6 axis may represent a putative module linking post-transcriptional regulation, granulosa-cell function and follicular development.

Unlike the laying-maintenance modules associated with CPEB1 and BMP6, the FOXO3-related ceRNA network was primarily associated with energy sensing, cellular quiescence and follicular atresia. FOXO3 may represent an important downstream regulatory node within this network. Our data showed that novel_circ_002435 and novel_circ_007511 were more abundant in NG, potentially enabling them to compete more effectively for miR-135-x/z. This may reduce miR-135-x/z-mediated repression of FOXO3, allowing FOXO3 to maintain a relatively high expression level during the nesting period. Pearson correlation analysis further showed that both circRNAs were negatively correlated with miR-135-x/z but positively correlated with FOXO3, whereas miR-135-x/z was negatively correlated with FOXO3. FOXO3 is an important transcription factor downstream of the PI3K-Akt signalling pathway. PI3K-Akt signalling controls follicular quiescence and growth by regulating FOXO3 phosphorylation and nucleocytoplasmic translocation. When Akt signalling is active, FOXO3 is phosphorylated and translocated to the cytoplasm, resulting in the suppression of its transcriptional activity. By contrast, increased nuclear FOXO3 activity favours the maintenance of cellular quiescence [41]. FOXO3 expression is markedly increased in granulosa cells from early atretic follicles in porcine ovaries and closely corresponds to regions of granulosa-cell apoptosis [42]. In chicken follicles and granulosa cells, FOXO3 can activate apoptosis-related molecules, indicating that it may participate in follicular atresia [43]. In hen granulosa cells, FSH triggers FOXO3/4 phosphorylation and their export from the nucleus, thereby stimulating cell growth while limiting apoptotic activity. These findings implicate the intracellular localisation of FOXO proteins in follicle development [44]. Therefore, the novel_circ_002435/novel_circ_007511-miR-135-x/z-FOXO3 axis may be associated with reduced follicular growth and atresia during the nesting period through links to cellular quiescence, stress responses and granulosa-cell apoptotic programmes; and direct functional evidence is required to test this interpretation.

In addition to the core ceRNA regulatory axes described above, other DE mRNAs further reflected changes in signal transduction, ion homeostasis and stress adaptation in the ovary during the nesting period. Expression of GABRA2, HRH3, CACNA1C and FSTL1 was higher in nesting ovaries under the nominal screening criteria. GABRA2 and HRH3 contribute to γ-aminobutyric acid and histamine pathways, respectively. These signalling systems involve ligand-gated ion transport, G-protein-mediated receptor activity and the maintenance of cellular calcium balance. Members of the GABA receptor family may participate in regulating follicular development and laying performance. DE genes in chicken ovarian follicles across laying stages are also associated with pathways including neuroactive ligand-receptor interaction and calcium signalling [45]. Local ovarian GABA-receptor signalling is associated with steroidogenesis, follicular development and granulosa-cell apoptosis [46]. Therefore, the upregulation of GABRA2 during the nesting period may reflect an adaptive ovarian response to local signalling changes through neuroendocrine regulation.

The protein encoded by CACNA1C forms part of an L-type voltage-gated calcium channel and contributes to membrane Ca^2+^ transport, intracellular calcium balance and activation of downstream signalling. During follicular selection, avian granulosa cells exhibit marked changes in genes associated with ion signalling, hormonal responses and cell differentiation [47]. CACNA1C has also been identified among the genes associated with GnRH signalling and with ovarian functional changes between the laying and incubation periods in hens [48]. In the present study, CACNA1C was associated with GnRH secretion and calcium signalling, suggesting that it may participate in adjustments to ovarian calcium signalling and cellular responses during the nesting period. FSTL1 is a secreted glycoprotein that is mainly involved in cell-surface receptor signalling, growth-factor responses and cellular responses to chemical stimuli. FSTL1 is closely associated with gene-expression changes and local tissue responses in preovulatory granulosa cells [49]. From the perspectives of receptor signalling, ion transport and secreted-factor regulation, these genes may represent additional molecular features associated with the ovarian functional transition predicted by the putative ceRNA regulatory axes.

To further examine the relationships between the proposed ceRNA regulatory axes and reproductive endocrine status, we analysed correlations between the expression levels of core circRNAs, miRNAs and mRNAs and the serum concentrations of E2, AMH, P4, LH, FSH and PRL. Overall, the selected RNAs showed two opposing hormone-correlation patterns. Components with higher expression in LLG were generally positively correlated with E2, AMH and P4 as well as LH and FSH but negatively correlated with PRL. Components with higher expression in NG showed the reverse pattern. This pattern was broadly consistent with the endocrine characteristics of the nesting period, including increased PRL concentrations, decreased E2, P4 and LH concentrations, no significant differences in AMH or FSH concentrations, and reduced follicular activity and ovarian steroidogenic capacity. CPEB1 and BMP6, which are involved in oocyte maturation and granulosa-cell function, and their associated circRNAs, including novel_circ_001280 and novel_circ_000095, were positively correlated with E2, AMH, P4, LH and FSH but negatively correlated with PRL. By contrast, FOXO3, which is associated with cellular quiescence, stress responses and follicular atresia, together with the nesting-upregulated novel_circ_002435 and novel_circ_007511, was positively correlated with PRL but negatively correlated with E2, AMH, P4, LH and FSH [50]. These results suggest that the CPEB1- and BMP6-related regulatory axes may reflect links to follicular development and ovarian steroidogenic status, whereas the FOXO3-related regulatory axis may be more closely involved in ovarian stress responses and regression during the nesting period. Correlation analysis alone is insufficient to fully establish direct relationships between hormonal changes and the proposed ceRNA regulatory axes. Nevertheless, these findings provide a basis for understanding the coordinated changes in non-coding RNA networks and ovarian endocrine status.

However, this study has several limitations. Only three biological replicates per stage were used for RNA sequencing which limited statistical power and the robustness of expression and correlation analyses. Correlations across six samples may partly reflect between-stage differences rather than direct regulatory interactions. Across the complete result tables, 1 circRNA, 2 miRNAs and 195 mRNAs met FDR < 0.05 and |log_2_FC| ≥ 1. None of the 11 circRNA components included in the selected ceRNA networks met these criteria. The networks derived from the nominal screening criteria should therefore be regarded as exploratory hypotheses. The target relationships were predicted computationally, and functional perturbation or direct interaction experiments were needed. RT-qPCR used the same biological samples of RNA sequencing and therefore supports cross-platform consistency rather than independent-cohort validation. The proposed ceRNA relationships remain preliminary and need validation in additional animals and functional experiments.

## 5. Conclusions

In summary, ovarian circRNA, miRNA and mRNA profiles were characterised in Wanxi white geese during LLG and NG, and the resulting datasets were integrated to propose an exploratory ceRNA framework. Pathway analysis linked the genes hosting DE circRNAs to membrane and junctional structures, small GTPase-mediated signal transduction, cell adhesion, ECM–receptor interaction, adherens junction, focal adhesion and metabolic regulation. In contrast, targets predicted for DE miRNAs showed enrichment in the AMPK, mTOR, TGF-β and Hippo pathways, as well as oocyte meiosis. Three putative relationships were prioritised from the integrated analysis. In the novel_circ_001280–miR-1-y–CPEB1 and novel_circ_005385–miR-187-x–BMP6 relationships, the circRNAs and target mRNAs were higher during late laying, whereas the corresponding miRNAs were higher during nesting; these relationships may be associated with maternal mRNA translational activation and meiotic maturation on the one hand, and with granulosa-cell BMP signalling on the other. By contrast, in the novel_circ_002435/novel_circ_007511–miR-135-x/z–FOXO3 relationship, the circRNAs and FOXO3 were higher during nesting, whereas miR-135-x and miR-135-z were higher during late laying; this relationship was associated with expression patterns related to cellular quiescence, stress responses and follicular atresia. The expression of these components was correlated with circulating E2, P4, AMH, LH, FSH and PRL concentrations. Because the proposed relationships have not yet been experimentally verified, they should be regarded as predictions. Nevertheless, these observations provide an initial basis for functional testing and warrant further evaluation in future studies of broodiness and laying performance in waterfowl.

## Figures and Tables

**Table 1:** Quality-control summary of the small-RNA sequencing libraries.

Sample	Raw Reads	Clean Reads	3’adapter Null	Insert Null	5’adapter Contaminants	polyA	Clean Tags
NG-1	13,010,907	12,863,090	3791	177,181	9558	96	12,537,829
NG-2	12,579,180	12,429,192	12,803	11,589	6111	98	12,305,559
NG-3	13,018,681	12,878,812	9736	6197	2151	39	12,813,377
LLG-1	13,661,232	13,513,634	6198	9904	3326	59	13,423,841
LLG-2	13,449,192	13,305,883	6174	9732	3674	67	13,187,559
LLG-3	13,547,338	13,424,244	97,168	7742	3410	36	13,250,083

**Table 2:** Numbers of known and novel miRNAs identified and their tag abundance in each sample.

Sample	Total Clean-Tag Abundance	Known miRNAs				Novel miRNAs			
		No. Identified	Unique Tags	Tag Abundance	Proportion (%)	No. Identified	Unique Tags	Tag Abundance	Proportion (%)
NG-1	12,537,829	439	18,332	11,982,369	95.57	88	190	3093	0.02
NG-2	12,305,559	419	20,861	11,546,298	93.83	107	259	5361	0.04
NG-3	12,813,377	391	20,657	12,464,142	97.27	85	205	2551	0.02
LLG-1	13,423,841	410	21,201	12,626,162	94.06	104	250	5645	0.04
LLG-2	13,187,559	410	21,243	12,470,317	94.56	102	255	5229	0.04
LLG-3	13,250,083	398	20,723	12,824,597	96.79	96	230	3557	0.03

## Data Availability

The transcriptome sequencing data were deposited in the Sequence Read Archive (SRA) database (https://www.ncbi.nlm.nih.gov/sra (accessed on 28 September 2026)) of NCBI under the BioProject accession numbers PRJNA1513041 and SAMN62403236 to SAMN62403241, and the SRA project accession numbers SRR40170502 to SRR40170513. The previously published mRNA dataset used in this study is available in the NCBI SRA under run accessions SRR30688733–SRR30688738.

## Supplementary Materials

The following supporting information can be downloaded at https://www.mdpi.com/article/10.3390/genes17101227/s1: Table S1, Primers used for quantitative real-time PCR in this study; Table S2, Quality-control summary of the circRNA sequencing libraries; Table S3, Key mRNAs in the ceRNA network associated with egg laying and nesting in Wanxi white geese. Table S4, circRNAs, miRNAs, and mRNAs meeting FDR < 0.05 and |log_2_FC| ≥ 1 between the late-laying and nesting stages in Wanxi white geese; Table S5, All circRNAs associated with egg laying and nesting in Wanxi white geese; Table S6, All miRNAs associated with egg laying and nesting in Wanxi white geese; Table S7, All mRNAs associated with egg laying and nesting in Wanxi white geese; Table S8, Selected circRNAs, miRNAs, and mRNAs in the ceRNA network associated with egg laying and nesting in Wanxi white geese.

## Abbreviations

The following abbreviations are used in this manuscript:

AKT	protein kinase B
AMPK	AMP-activated protein kinase
AMH	anti-Müllerian hormone
bp	base pair
BSJ	back-splice junction
cDNA	complementary DNA
ceRNA	competing endogenous RNA
cGMP	cyclic guanosine monophosphate
circRNA	circular RNA
DE	differentially expressed
dUTP	deoxyuridine triphosphate
E2	oestradiol
EB	elution buffer
ECM	extracellular matrix
ELISA	enzyme-linked immunosorbent assay
FPKM	fragments per kilobase of transcript per million mapped reads
FSH	follicle-stimulating hormone
GABA	gamma-aminobutyric acid
GC	guanine-cytosine
GO	Gene Ontology
HPO	hypothalamic–pituitary–ovarian
kb	kilobase
KEGG	Kyoto Encyclopedia of Genes and Genomes
LH	luteinising hormone
LLG	late-laying group
lncRNA	long non-coding RNA
MAPK	mitogen-activated protein kinase
miRNA	microRNA
mRNA	messenger RNA
mTOR	mechanistic target of rapamycin
NCBI	National Center for Biotechnology Information
NG	nesting group
nt	nucleotide
P4	progesterone
PAGE	polyacrylamide gel electrophoresis
PCR	polymerase chain reaction
PI3K	phosphoinositide 3-kinase
PKG	cGMP-dependent protein kinase
PPAR	peroxisome proliferator-activated receptor
PRL	prolactin
Q30	Phred quality score of 30
RNA	ribonucleic acid
RNA-seq	RNA sequencing
RPM	reads per million mapped reads
rRNA	ribosomal RNA
RT-qPCR	reverse transcription quantitative polymerase chain reaction
scRNA	small cytoplasmic RNA
snoRNA	small nucleolar RNA
snRNA	small nuclear RNA
SRA	Sequence Read Archive
TGF-β	transforming growth factor beta
TPM	transcripts per million
tRNA	transfer RNA
UNG	uracil-N-glycosylase
