# Supplementary material for: Integrated Ovarian circRNA, miRNA and mRNA Profiling Identifies Candidate ceRNA Relationships Associated with Late-Laying and Nesting Stages in Wanxi White Geese
Source: Genes (Basel). 2026 Oct 2;17(10):1227. doi: 10.3390/genes17101227 (PMC13704495; doi:10.3390/genes17101227)
Supplement: Supplementary file 1 [file genes-17-01227-s001.zip › Supplementary Tables S1-S2.pdf]

Integrated Ovarian circRNA, miRNA and mRNA Profiling Identifies Candidate ceRNA Relationships Associated with Late-Laying and Nesting Stages in Wanxi White Geese

Table S1: primers used for Quantitative Real-Time PCR in this study

Gene Name	Primer sequences (5'-3')
CPEB1	F:TACAAGAACCCTGTCTACTCCT<br>R:TGTTTGCCATCCTTACCAG
BMP6	F:CCCAGGCTCAGGATGTTT<br>R:TGTGCGTTGAGTGGAAATG
Foxo3	F:CTGGATGCGTGGACAGATT<br>R:CGGAGCGTCATCGTCTTG
GABRA2	F:GGAAGAAGTCAGTGGCTCA<br>R:GGGAAGTCCTCCAAGTGC
HRH3	F:GTGCCCTATGTGCTGACG<br>R:CGATGTTGAAGACCGAGGA
novel_circ_005385	F:GTTCCCCAATGCCGTCTTTT<br>R:ATGCCACGATGTTTCACAGC
novel_circ_000230	F:ATGCAGGATTCAACCATGGC<br>R:TTTTGCAGCTTGGGTTTGGA
novel_circ_001280	F:CAATCGACAGCTGCATGACA<br>R:CTCCAGATTTCAGCTTGGCA
novel_circ_002435	F:CACCGCAGTCAGAAGCTTTC<br>R:TCAGCAACACGGATCAGACA
novel_circ_001139	F:CAGTCCCGTACCCAGAAGAA<br>R:CTCTGAAGACCCTCCCACTC
novel_circ_007511	F:CAGCTCACCCAGACCTACC<br>R:TTAGTCAACTAGCAGGCGGG
β-actin	F:GCACCCAGCACGATGAAAAT<br>R:GACAATGGAGGGTCCGGATT
miR-1-y	RT:GTCGTATCCAGTGCAGGGTCCGAGGTA<br>TTCGCACTGGATACGACTCTCTT<br>F：GCGCGCGGGAAGAAAG<br>R：AGTGCAGGGTCCGAGGTATT
miR-187-x	RT:GTCGTATCCAGTGCAGGGTCCGAGGTA<br>TTCGCACTGGATACGACCTCCCT<br>F：CGCGGCACAACACAGGAC<br>R：AGTGCAGGGTCCGAGGTATT

Gene Name	Primer sequences (5’-3’)
miR-215-y	RT:GTCGTATCCAGTGCAGGGTCCGAGGTA<br>TTCGCACTGGATACGACTTGGCC<br>F：CGCGCGCGCCGCACGA<br>R：AGTGCAGGGTCCGAGGTATT
miR-31-x	RT:GTCGTATCCAGTGCAGGGTCCGAGGTA<br>TTCGCACTGGATACGACGCTTGC<br>F：CGCGCGAGGCAAGAGG<br>R：AGTGCAGGGTCCGAGGTATT
miR-72-x	RT:GTCGTATCCAGTGCAGGGTCCGAGGTA<br>TTCGCACTGGATACGACGCTTGC<br>F：CGCGCGAGGCAAGAGG<br>R：AGTGCAGGGTCCGAGGTATT
miR-135-x	RT:GTCGTATCCAGTGCAGGGTCCGAGGTA<br>TTCGCACTGGATACGACTCCTGG<br>F：GCGCGCGCGCGAGGCA<br>R：AGTGCAGGGTCCGAGGTATT
miR-135-z	RT:GTCGTATCCAGTGCAGGGTCCGAGGTA<br>TTCGCACTGGATACGACTTCCTG<br>F：CGCGCGCGCGAGGCAC<br>R：AGTGCAGGGTCCGAGGTATT
U6	RT:AACGCTTCACGAATTTGCGT<br>F：CTCGCTTCGGCAGCACA<br>R：AACGCTTCACGAATTTGCGT

Table S2. Quality-control summary of the circRNA sequencing libraries

Sample	Raw Reads	Clean Reads	Mapped Reads	Mapped Rates(%)	Clean Q30(%)	GC Content (%)
NG-1	96,749,722	95,041,452	47,782,164	50.64	90.20	62.37
NG-2	92,608,232	91,815,572	65,891,492	72.13	91.93	54.12
NG-3	93,112,208	92,282,652	64,189,644	69.93	92.33	54.13
LLG-1	97,975,888	97,144,090	70,001,126	72.41	92.84	54.97
LLG-2	88,386,414	87,632,922	62,810,302	72.16	92.26	54.38
LLG-3	99,116,494	98,058,298	64,614,834	66.33	92.03	56.98
